# Anterior Cruciate Ligament Injury Prevention Programs in Welsh Netball: A Survey of Knowledge, Implementation and Barriers Amongst Players and Coaches

**DOI:** 10.7759/cureus.60405

**Published:** 2024-05-16

**Authors:** Wahid Abdul, Isabel Moore, Angus Robertson

**Affiliations:** 1 Trauma and Orthopaedics, University Hospital of Wales, Cardiff, GBR; 2 Sports and Exercise Medicine, Cardiff Metropolitan University, Cardiff, GBR; 3 Orthopaedic Surgery, University Hospital of Wales, Cardiff, GBR

**Keywords:** coaches, players, netball, injury prevention program, anterior cruciate ligament (acl)

## Abstract

Background

Anterior cruciate ligament (ACL) injury prevention programs can reduce injury risk in various sports. The perception of ACL injury prevention programs amongst professional netball players and coaches has not been studied.

Purpose

The aim of this study was to determine (1) the level of awareness and experience of ACL injury prevention programs, (2) the use of ACL injury prevention programs, and (3) barriers and potential facilitators to implementing a sustainable ACL injury prevention program in netball.

Materials and methods

Female netball players representing Welsh senior and under-21 teams and elite and amateur coaches were invited electronically to participate in this web-based cross-sectional observational study between 1^st^ May and 31^st^ July 2021. Information on ACL injury susceptibility and seriousness, knowledge, experience, and implementation of ACL injury prevention programs were ascertained.

Results

Twenty-eight players (78%) and 29 coaches (13%) completed the questionnaire. Seventeen (61%) players and 15 (52%) coaches reported that female athletes were at greater risk of sustaining ACL injuries. Over 90% of respondents identified netball as high-risk, whilst 89% (n=25) of players and 76% (n=22) of coaches reported these injuries to be preventable. Only two (7%) players and six (21%) coaches utilised an ACL injury prevention program with a lack of time and engagement from coaches and players identified. The majority of respondents indicated that their club has neither promoted, advocated the use nor demonstrated exercises for ACL injury prevention. Over 90% of respondents would utilise an ACL injury prevention program if it minimised players' risk with appropriate information and demonstration of exercises.

Conclusion

This study highlights limited knowledge of female athletes’ increased susceptibility to ACL injuries amongst players and coaches with a lack of communication and education on ACL injury prevention programs between sporting associations, coaches, and players. However, the results demonstrate willingness amongst both players and coaches to implement an ACL injury prevention program in netball.

## Introduction

Netball involves rapid acceleration, deceleration, pivoting, change of direction, running, and jump landing. It is played across 70 countries with over 20 million participants worldwide with an injury rate of 11.3-14 and 19.35 per 1000 playing hours for recreational and elite athletes, respectively [1]. Australian netball reported the prevalence of anterior cruciate ligament (ACL) injuries to be between 17.2 and 22.4% [2]. Two distinct patterns of ACL injuries in netball have been identified, ‘indirect contact’ and ‘noncontact’, with the latter accounting for 80% of ACL injuries. Approximately 83.3% of ‘noncontact’ ACL injuries in netball occur during landing secondary to knee abduction collapse [3,4]. The ‘footwork rule’ restricts players to a one-step landing after catching the ball thus players pivot on the touchdown foot prior to releasing the ball [5]. This one-step landing creates a high vertical ground reaction force of up to 3.5-5.7 of body weight with or without the ball causing shear, compression, and rotational forces on the lower limb [6].

According to the United Kingdom National Ligament Registry (NLR) report published in 2022, 86% of ACL injuries were attributed to sporting activities including football (47.8%), skiing (12.2%), rugby (11.8%), other (6.2%) and netball (5.3%). However, when analysing the number of ACL injuries sustained in females who underwent surgery, netball (n=653) was only second to snow skiing (n=1,068) followed by football (n=454) [7].

Short- and long-term consequences of ACL injuries include lower return-to-play rates in females than males under 25 years of age (39% vs 52%) and 26-35 years of age (18% vs 36%) along with a four-fold increased risk of post-traumatic osteoarthritis [8,9].

Risk factors for ACL injuries can be classified into extrinsic (weather, playing surface, footwear, and level of competition) and intrinsic (anatomical (narrow intercondylar notch width), biomechanical (knee abduction collapse during landing), neuromuscular (increased quadriceps-to-hamstring ratio), hormonal and sex) [10]. Recent epidemiological studies have reported a rapid increase in ACL injuries among females compared to males (34% versus 13%) (p<0.01) [11]. Compared to their male counterparts, female athletes have a 2-9 times greater risk of sustaining noncontact ACL injuries, with younger females participating in cutting, jumping, and pivoting manoeuvres demonstrating 2-4 times greater risk [12].

Research around modifiable risk factors such as biomechanical and neuromuscular risks has led to the development of various ACL injury prevention programs including the Cincinnati Sportsmetric Program, Henning Program, Prevent Injury Enhance Performance (PEP), Footy First, The FIFA Medical and Research Centre (F-MARC), Federation International de Football Association (FIFA) 11 and FIFA 11+ [13-15]. These multicomponent injury prevention programs focus on muscle strengthening and recruitment patterns, landing biomechanics, deceleration, proprioception, and plyometrics by incorporating agility, balance, mobility, plyometrics, running, and strength activities [16]. Incorporation of F-MARC, FIFA 11, and FIFA 11+ in football has led to an overall risk reduction of 34% (RR= 0.66) for all injuries and 29% (RR=0.71) for lower limb injuries [17]. Furthermore, universal neuromuscular preventative training programs have been demonstrated to reduce the incidence of ACL injuries among young athletes from 3% to 1.1% per season with an average saving of $100 per player per season [18].

Various sports, including football, field hockey, and volleyball, have evaluated the perception of ACL injury prevention programs among players and coaches [19-22]. However, the perception of ACL injury prevention programs among female netball players and coaches has not been studied. The primary aim of this study was to investigate the awareness and experience of ACL injury prevention programs amongst elite Welsh netball players and elite and amateur regional coaches. The secondary aim was to identify potential barriers and solutions for implementing a well-maintained ACL injury prevention program that can be utilised across Welsh netball to mitigate the risk of sustaining ACL injuries.

## Materials and methods

Study design

This cross-sectional observational study was undertaken using the Checklist for Reporting Results of Internet E-Surveys (CHERRIES) checklist [23]. Ethical approval was granted by Cardiff School of Sport and Health Sciences under Cardiff Metropolitan University Research Ethics Framework [PGT-3791].

Survey design

A web-based survey administered through Qualtrics XM 2021 (Qualtrics International Inc., Utah), which is General Data Protection Regulation (GDPR) compliant and provided by Cardiff Metropolitan University, was devised to collect information on netball players and coaches’ knowledge, implementation, and barriers to ACL injury prevention programs. Question development by the primary author was guided by the Health Belief Model (HBM) constructs, and the Reach Effectiveness Adoption Implementation Maintenance (RE-AIM) framework [24,25]. Pilot testing by authors allowed critical appraisal of questions to ascertain clarity, comprehension, and appropriateness. This open survey consisted of 34 questions for players and 29 questions for coaches including, demographics, ACL injury susceptibility and seriousness, knowledge, experience, and implementation of ACL injury prevention programs (Appendix A). Questions that sought opinion-based answers were either yes/no or collected on a five-point Likert scale with 1 representing ‘strongly agree’ and 5 representing ‘strongly disagree’. In certain questions, participants were able to choose multiple options.

Study participants

During the 2020-2021 season, 23 players represented the senior squad, and 19 players represented the under-21 squad. Six players were excluded as they were under 18 years. There were five national coaches (three senior and two under-21) and 214 affiliated members with a coaching qualification from 112 regional clubs. Coaching credentials included two post-graduate qualifications and a UK Coaching Certificate (UKCC) for netball including level 1 (n=9), level 2 (n=18), and level 3 (n=2). Players and coaches were invited electronically by the Performance Lead at Welsh Netball outlining the purpose of the survey and survey link which was accessible from 1st May to 31st July 2021. The first page described the purpose of the study followed by an electronic written consent form that participants completed. Once respondents had submitted the survey, they could not respond again nor amend it as data was automatically stored. All responses were voluntary and anonymous. An email reminder was sent fortnightly with no incentives offered.

Data analysis

Data was extracted from Qualtrics with only complete questionnaires analyzed. In view of the small sample size and lack of variability, 5-point Likert scales were collapsed into three-point scales: ‘strongly agree/agree’, ‘neither agree nor disagree’ and ‘disagree/strongly disagree’. Descriptive statistics are presented as mean and absolute numbers. Cross-tabulation tables for players and coaches including frequency and percentages were displayed and compared using the Chi-squared test via GraphPad Prism version 9 (GraphPad Software, San Diego, CA) (Appendix B). A p-value of <0.05 was set for statistical significance.

## Results

Twenty-eight players (78%) completed the questionnaire, 71% (n=20) senior players and 29% (n=8) under-21 players, with most respondents aged 18-21 years (n=12, 43%) playing netball for 6-10 years (n=12, 43%). Twenty-nine (13%) coaches completed the questionnaire, 27 amateur coaches (93%) and two professional coaches (7%). Nine coaches had less than five years of experience whilst 8 had over 21 years of experience.

Perceptions of ACL injury susceptibility and injury seriousness

**Figure 1:**
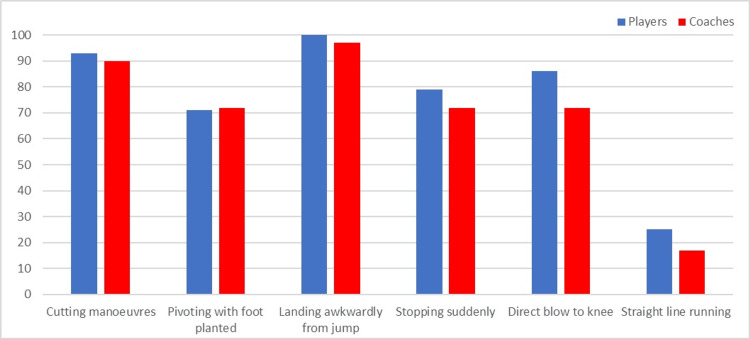
The proportion (%) of players (blue bars) and coaches (red bars) reporting each perceived mechanism of an anterior cruciate ligament injury. Nb. Respondents could choose multiple options; the data has been represented as %.

The most common perceived mechanisms of ACL injuries for players and coaches were ‘landing awkwardly from a jump’ and ‘cutting manoeuvres’ (Figure 1).

The most common risk factors for ACL injury for players and coaches were ‘previous ACL injury’, ‘fatigue’, ‘jump landing’ and ‘inappropriate footwear’ (Table 1).

**Table 1:** The proportion (%) of players and coaches reporting risk factors for anterior cruciate ligament injury. Nb. Respondents could choose multiple options; the data has been represented as n, %. ACL: Anterior cruciate ligament

Risk factors for ACL injury	Players (n=28)	Coaches (n=29)
Previous ACL injury	25 (89%)	27 (93%)
Fatigue	25 (89%)	15 (52%)
Jump landing	23 (82%)	27 (93%)
Playing surface	23 (82%)	21 (72%)
Inappropriate footwear	19 (68%)	19 (66%)
Female sex	13 (46%)	10 (35%)
Hormonal	11 (39%)	6 (21%)
Genetics	10 (36%)	10 (35%)
Weather	9 (32%)	11 (40%)
Flexibility	8 (29%)	9 (31%)
Age	7 (25%)	7 (24%)
High Body Mass Index	5 (18%)	8 (28%)
None	1 (4%)	0 (0%)

Three (11%) players sustained an ACL injury requiring surgery with a mean return-to-play of 8.7 months (range 6-11 months). Eighty-six percent (n=24) of players and 55% (n=16) of coaches were aware of a player who had sustained a season-ending ACL injury.

Table 2 illustrates that 61% (n=17) of players and 52% (n=15) of coaches identified female athletes at greater risk of sustaining an ACL injury whilst over 90% of respondents identified netball players at greater risk. A similar proportion of players and coaches identified the burden of ACL injury on career (86%, n=24 vs 94%, n=27), physical disability (86%, n=24 vs 90%, n=26) and quality of life (86%, n=24 vs 83%, n=24). A greater proportion of coaches (72%, n=21) than players (64%, n=18) believed that ACL injuries have negative impact on team performance.

**Table 2:** Respondent’s perceptions of ACL injury susceptibility and injury seriousness *RE-AIM framework: E: effectiveness, A: adoption, I: implementation, M: maintenance †P: players, C: coaches Nb. The data has been represented as %, n. p<0.05 considered statistically significant. ACL: Anterior cruciate ligament

Statement	HBM Construct	RE-AIM* Dimension(s)	Agree % (n)		Neither Agree/ Disagree % (n)		Disagree % (n)		X^2^	p-value
			P	C	P	C	P	C		
Female athletes are at increased risk for sustaining ACL injury than male athletes	Perceived susceptibility	A, M	61 (17)	52 (15)	25 (7)	41 (12)	14 (4)	7 (2)	2.09	0.352
Netball players are at a high risk of ACL injury	Perceived susceptibility	A, M	93 (26)	97 (28)	7 (2)	3 (1)	0 (0)	0 (0)	0.39	0.532
ACL injuries can shorten a Netball player’s career	Perceived severity	A, M	86 (24)	94 (27)	14 (4)	3 (1)	0 (0)	3 (1)	2.96	0.228
ACL injuries can cause physical problems later in life	Perceived severity	A, M	86 (24)	90 (26)	14 (4)	10 (3)	0 (0)	0 (0)	0.21	0.650
ACL injuries have a negative impact on Netball player’s quality of life	Perceived severity	A, M	86 (24)	83 (24)	11 (3)	10 (3)	3 (1)	7 (2)	0.32	0.854
Participating with an ACL injury can have a negative impact on team performance	Perceived severity	A, M	64 (18)	72 (21)	25 (7)	21 (6)	11 (3)	7 (2)	0.49	0.783

Perceptions of ACL injury prevention programs

According to players and coaches, stakeholders responsible for delivering ACL injury prevention programs include physiotherapists (79%, n=22 vs 17%, n=5), fitness coaches (11%, n=3 vs 21%, n=6) and head coach (7%, n=2 vs 35%, n=10), respectively (X2=22.83, p=0.0004). Compared to coaches (76%, n=22), a greater proportion of players (89%, n=25) indicated that ACL injuries sustained during netball were preventable, with exercises preventing ACL injuries requiring variation and progression over time including eccentric muscle strengthening and cutting manoeuvres along with a warm-up and cool-down jogs (Table 3).

**Table 3:** Players and coaches’ perceptions of ACL injury prevention programs *RE-AIM framework: E: effectiveness, A: adoption, I: implementation, M: maintenance †P: players, C: coaches Nb. The data has been represented as %, n. p<0.05 considered statistically significant. ACL: Anterior cruciate ligament

Statement	HBM Construct	RE-AIM* Dimension(s)	Agree % (n)		Neither Agree/ Disagree % (n)		Disagree %(n)		X^2^	p-value
			P	C	P	C	P	C		
It is possible to prevent some ACL injuries in netball	Perceived benefit	A, E	89 (25)	76 (22)	7 (2)	24 (7)	4 (1)	0 (0)	3.95	0.139
Exercises scientifically proven to prevent ACL injuries should be performed by netball players	Perceived benefit	A, I, M	100 (28)	100 (29)	0 (0)	0 (0)	0 (0)	0 (0)		
Exercises scientifically proven to prevent ACL injuries should be incorporated into clubs’ training guidelines	Cues to action	A, I, M	100 (28)	97 (28)	0 (0)	3 (1)	0 (0)	0 (0)	0.98	0.322
Exercises to prevent ACL injuries should be varied and progressed over time	Cues to action	A, I, M	93 (26)	79 (23)	0 (0)	21 (6)	7 (2)	0 (0)	8.17	0.017
Exercises involving balance training can prevent ACL injuries	Perceived benefit	E, A, I	86 (24)	87 (25)	14 (4)	10 (3)	0 (0)	3 (1)	1.15	0.564
Exercises involving controlled jumping and landing can prevent ACL injuries	Perceived benefit	E, A, I	93 (26)	97 (28)	7 (2)	0 (0)	0 (0)	3 (1)	3.06	0.217
Exercises involving eccentric muscle strengthening can prevent ACL injuries	Perceived benefit	E, A, I	86 (24)	66 (19)	14 (4)	34 (10)	0 (0)	0 (0)	3.14	0.077
Exercises involving cutting manoeuvres can prevent ACL injuries	Perceived benefit	E, A, I	82 (23)	66 (19)	11 (3)	28 (8)	7 (2)	7 (2)	2.64	0.268
A warm-up jog/run can prevent ACL injuries	Perceived benefit	E, A, I	54 (15)	48 (14)	46 (13)	28 (8)	0 (0)	24 (7)	8.21	0.017
A cool-down jog/run can prevent ACL injuries	Perceived benefit	E, A, I	46 (13)	45 (13)	46 (13)	31 (9)	7 (2)	24 (7)	3.49	0.175

Eighteen (64%) players and 23 (79%) coaches indicated that exercises preventing ACL injuries should be incorporated as part of training. Approximately 54% (n=15) of players indicated an ideal duration for warm-up session of 20-30 minutes whilst 79% (n=20) of coaches reported 10-20 minutes as the ideal duration (X2=10.30, p=0.016).

Experience with ACL injury prevention programs

Sixty-two percent of coaches (n=18) reported that coaching courses undertaken did not discuss the risk of ACL injuries in netball. Only three players (11%) attended training or had been directed to resources on ACL injury prevention whilst four players (14%) had exercises demonstrated to them. The majority of players and coaches indicated that their netball club has neither promoted awareness of ACL injury, advocated the use nor demonstrated exercises for ACL injury prevention (Table 4).

**Table 4:** Players and coaches experience with ACL injury prevention programs *RE-AIM framework: E: effectiveness, A: adoption, I: implementation, M: maintenance †P: players, C: coaches Nb. The data has been represented as %, n. p<0.05 considered statistically significant. ACL: Anterior cruciate ligament; IPP: injury prevention program

Statement	HBM Construct	RE-AIM* Dimension(s)	Agree % (n)		Neither Agree/ Disagree % (n)		Disagree % (n)		X^2^	p-value
			P	C	P	C	P	C		
Netball club has promoted awareness about risk of ACL injuries amongst Netball players, either in meetings, emails, or by other means	Perceived benefit	A, I, M	29 (8)	28 (8)	14 (4)	31 (9)	57 (16)	41 (12)	2.48	0.290
Netball club has advocated use of an ACL IPP for my team, or directed me to resources to find information about such programs	Perceived benefit	A, I, M	29 (8)	21 (6)	14 (4)	31 (9)	57 (16)	48 (14)	2.33	0.313
Netball coach/club representative has demonstrated how to perform an ACL IPP to me or my team	Perceived benefit	A, I, M	21 (6)	14 (4)	11 (3)	24 (7)	68 (19)	62 (18)	2.83	0.242

Implementation of ACL injury prevention programs

Two (7%) players previously utilised an ACL injury prevention program with problems identified including lack of time (n=2), ideas (n=1), enthusiasm from coaches (n=1) and players (n=1). Six (21%) coaches previously employed an ACL injury prevention program with problems encountered including lack of time (n=5), enthusiasm from players (n=4), ideas for training drills (n=3), players deeming program as unnecessary (n=3), engagement from coaches (n=2), poor player attendance (n=2), coaches deeming program as unnecessary (n=1), drills not stimulating (n=1) and lack of equipment (n=1).

Among those not currently using an ACL injury prevention program, a greater proportion of players compared to coaches would utilise these programs instead of a warm-up (81%, n=22 vs 65%, n=15). The majority (83%, n=19) of coaches were not implementing these programs as they were unfamiliar with the exercises, with 96% (n=22) of coaches indicating a willingness to employ these programs with access to information and demonstration of exercises. All players would utilise an ACL injury prevention program if exercises were demonstrated (Table 5).

**Table 5:** Implementation of ACL injury prevention programs *RE-AIM framework: E: effectiveness, A: adoption, I: implementation, M: maintenance †P: players, C: coaches Nb. The data has been represented as %, n. p<0.05 considered statistically significant. ACL: Anterior cruciate ligament; IPP: injury prevention program

Statement	HBM Construct	RE-AIM* Dimension(s)	Agree % (n)		Neither Agree/ Disagree %		Disagree % (n)		X^2^	p-value
			P	C	P	C	P	C		
I would consider using IPP if it could be used in place of warm up	Cues to action	A, I, M	81 (21)	65 (15)	8 (2)	13 (3)	12 (3)	22 (5)	1.52	0.467
The reason I do not use ACL IPP with my team is because I do not know the exercises	Perceived susceptibility	A, M	69 (18)	83 (19)	12 (3)	13 (3)	19 (5)	4 (1)	2.52	0.284
I would consider using an ACL IPP if I could access information about the exercises	Cues to action	A, M	96 (25)	96 (22)	0 (0)	4 (1)	4 (1)	0 (0)	2.02	1.07
I would consider using ACL IPP if somebody demonstrated the exercises properly	Cues to action	A, M	100 (26)	96 (22)	0 (0)	4 (1)	0 (0)	0 (0)	1.15	1.07

Knowledge of ACL injury prevention programs reducing risk of injury would persuade 93% (n=26) of players and 90% (n=26) of coaches to utilise these programs. Besides one, all players and coaches indicated an optimum duration of less than 20 minutes for ACL injury prevention programs.

## Discussion

To our knowledge, this is the first study to assess the perception of ACL injury prevention programs amongst netball players and coaches. Our study highlights the limited knowledge of female athletes’ increased susceptibility to ACL injuries with a lack of communication and awareness of these programs between players, coaches, and sporting associations. However, the results demonstrate a willingness amongst players and coaches to implement an ACL injury prevention program to minimise the risk of injury.

Perceptions of ACL injury susceptibility and injury seriousness

Approximately two-thirds (61%, n=17) of players and half (52%, n=15) of coaches reported that female athletes were more susceptible to sustaining ACL injuries than their male counterparts. Tanaka et al. surveyed 440 female athletes across 20 different sports with 85% of respondents recognising female athletes were at greater risk than male athletes [26]. With several studies reporting a 2-9 times greater risk of ACL injuries in female athletes, the findings of our study demonstrate a lack of awareness of injury susceptibility and seriousness in our cohort [8,12]. Reassuringly, over 90% of respondents considered netball as a high-risk sport in accordance with the findings of the NLR whereby netball was the second most common sport attributed to ACL injuries in females undergoing surgery [7]. These findings highlight the need for sporting associations to educate both athletes and coaches regarding female athletes’ increased susceptibility to ACL injuries.

A similar proportion of players and coaches recognised the negative impact of ACL injury including shortened career, problems later in life and quality of life, 83-94% respectively. These results were comparable to previous studies evaluating the perception of injury prevention in football amongst players and coaching staff [21,22]. Although netball and football are different sports, both involve jump landing, cutting and change-of-direction manoeuvres which can increase players’ susceptibility to ACL injury and thus findings can be comparable.

Negative associations between injury and individual and team performances have been well documented [27]. A study of male professional football players from the Spanish La Liga who had sustained moderate and major injuries demonstrated reduced playing time, participation and maximum speed achieved [27]. Another study of female and male Maltese football players reported that players had initially self-managed their injury and only reported injury depending on perceived pain severity. Stakeholders expressed that players were only injured when it hindered their performances [28]. The findings of this study further highlight the complexity of injury perception and reporting amongst players and coaches with coaches defining injury when it hinders players’ optimal performance and level of participation. In our study, a greater percentage of coaches (72%, n=21) than players (64%, n=18) recognised that participating with an ACL injury can have a negative impact on team performance. Our findings were comparable to a previous football study which reported 67% of players, compared to 100% of coaches, believed lower limb injuries had negative impact on team performance [22]. Another possible explanation for these findings can be attributed to coaches having a ‘birds-eye’ view of both individual and team performances from the sidelines. 

Perceptions of ACL injury prevention programs

According to our players, physiotherapists (79%, n=22) should be responsible for delivering the ACL injury prevention program whilst coaches indicated that the head coach (35%, n=10) should be ultimately responsible (X2=22.83, p=0.0004). Several studies have demonstrated coaches as pivotal intermediaries in the implementation of injury prevention programs with their motivation directly correlating with player motivation [4,21]. Therefore, it is imperative to adopt a collective approach with coaching staff and physiotherapists for the development, implementation, and long-term sustainability of injury prevention programs. This collaborative approach will not only ensure players engage with evidence-based ACL injury prevention programs but also foster good working relationships between stakeholders.

A greater percentage of players (89%, n=25) than coaches (76%, n=22) highlighted that netball injuries were preventable, emphasising the need to increase injury awareness, especially amongst coaches. All respondents indicated exercises scientifically proven to prevent ACL injuries should be performed by players, with all players and 97% (n=28) of coaches advocating incorporation of these exercises into training guidelines. These observations were comparable to football studies involving players, coaches and physiotherapists who reported injuries were preventable (90-100%), exercises scientifically proven to reduce injuries to be performed by players (98-100%) and incorporated in training guidelines (89-94%) [21,22]. Our findings demonstrate the willingness of netball players to engage with such programs to minimise the risk of ACL injuries.

The majority of players and coaches agreed that balance training (86%, n=24 vs 87%, n=25) and controlled jump landing (93%, n=26 vs 97%, n=28) can prevent ACL injuries. However, a greater proportion of players compared to coaches believed that eccentric strengthening (86%, n=24 vs 66%, n=19), cutting manoeuvres (82%, n=23 vs 66%, n=19) and warm-up jogs (54%, n=15 vs 48%, n=14) can prevent ACL injuries. Our results were comparable to a football study in which players, coaches and physiotherapists identified balance (94%), controlled jumping and landing (87%), eccentric muscle strengthening (85%) and cutting exercises (80%) to prevent football injuries [22]. Similarities identified can be attributed to similar movement patterns observed in both sports. However, differences observed in jump landing (95%) and eccentric strengthening (76%) in our study can be attributable towards different sporting demands, with jump landing more significant in netball and eccentric strengthening having superior importance in football due to greater distance covered and more sprinting involved. This may also provide an explanation why warm-up jogs (51%) were less significant in netball compared to football (82%).

Two-thirds (64%) of players and three-quarters (79%) of coaches reported that ACL injury prevention programs should be incorporated as part of training. Incorporation of such programs into training sessions will minimise exercise duplication and risk of injury from overtraining if such programs were to be separate from training.

Experience with ACL injury prevention programs

Sixty-two percent of coaches (n=17) reported that coaching courses undertaken did not discuss the risk of ACL injuries in netball. Our observations were comparable to another study in which 65.2% of football coaches reported courses attended did not discuss the risk of injury in football [20]. Only 11% (n=3) of our players attended training or been directed to resources on ACL injury prevention whilst 14% (n=4) had exercises demonstrated to them. The findings of these studies are concerning considering both sports are high risk for ACL injuries. Mandatory injury prevention training would empower coaching staff to deliver injury prevention programs starting from adolescent and amateur levels right through to adulthood.

The majority of the players and coaches highlighted that their netball club has neither promoted awareness of ACL injuries (57%, n=16 vs 41%, n=12), advocated use of ACL injury prevention programs (57%, n=16 vs 48%, n=14) nor directed them to resources (68%, n=19 vs 62%, n=18). Mawson et al. reported similar findings with coaches indicating that their clubs did not raise awareness (52.6%) nor advocated the use of injury prevention program (52.1%) [20]. A study published a decade ago demonstrated that utilisation of injury prevention programs was dependent upon education and communication from football associations and coaching courses [15]. These findings demonstrate that sporting clubs have a responsibility towards players and coaching staff to facilitate the provision and delivery of ACL injury prevention programs.

Implementation of ACL injury prevention programs

The proportion of players and coaches utilising ACL injury prevention programs in netball was low, 7% (n=2) vs 21% (n=6) respectively. Several barriers were identified including lack of time, ideas, and enthusiasm. The only study evaluating netball coaches’ feedback on the implementation of the Down-to-Earth injury prevention program reported program-related problems including lack of ideas, time, coaching skills, space, and equipment along with player-related factors including lack of motivation, awareness of the importance, attendance, and motivation [29]. A recent systematic review identified five potential barriers including, motivation, time constraints, skill of program facilitators, compliance, and cost [30]. Strategies to overcome these barriers and improve compliance include the incorporation of these programs into accredited coaching courses, disseminating educational resources and varying exercises to motivate players.

According to the HBM, the likelihood of an individual engaging in a health promotion and disease prevention program is dependent upon their perceived threat along with their belief in the effectiveness of the recommended health promotion [24]. Adherence to injury prevention programs depends upon sporting associations, coaches and players understanding the demographics susceptible to ACL injuries and the evidence-based injury risk-reduction benefits of such programs.

A high proportion of coaches reported not utilising these programs due to lack of awareness of exercises, whilst 96-100% of respondents would adopt such programs with relevant access to information and demonstration of exercises. Our observations were comparable to a football study whereby 84% of coaches indicated a willingness to implement an injury prevention program with access to information and demonstration of exercises [20]. Although these findings further highlight the lack of knowledge of such programs amongst coaching staff, both players and coaches are motivated to learn and implement these programs in netball.

In our study, the knowledge of ACL injury prevention programs reducing risk of injury would persuade 93% of players (n=26) and 90% of coaches (n=26) to perform these exercises. These findings were comparable to football studies whereby 84% of coaches advocated their use with the knowledge of injury reduction [20]. Another study of female hockey, football and volleyball athletes indicated willingness to implement ACL injury prevention programs if evidence demonstrated fewer risks of ACL injuries [19]. A recent study involving 440 female athletes across 20 different sports reported 89% of respondents favouring a preventive training program if it can prevent an ACL injury [26]. These findings further emphasise the importance of raising awareness of the injury risk-reduction benefits of these programs to improve compliance. 

Finally, the evidence for the clinical effectiveness of injury prevention programs is overwhelming. The Down-to-Earth program in junior netball highlighted benefits including improved landing technique (88%), strength, balance, coordination, and flexibility (83%) and injury risk reduction (79%) [29]. Incorporation of F-MARC, FIFA 11, and FIFA 11+ in football has led to an overall risk reduction of 34% (RR= 0.66) for all injuries and 29% (RR=0.71) for lower limb injuries [17]. Furthermore, incorporation of injury prevention programs in football can lead to an overall risk reduction of 34% (RR= 0.66) for all injuries and 29% (RR=0.71) for lower limb injuries [17]. Studies evaluating the efficacy of ACL injury prevention programs have demonstrated an average saving of $100 per player per season with a risk reduction from 3% to 1.1% per season, highlighting the financial and injury prevention benefits of such programs [18].

It is important to acknowledge several limitations of this study. As the sample size was small and the response rate only moderate, caution must be exercised when attempting to generalise findings to other settings. Due to the COVID-19 pandemic causing worldwide cessation of sports and remote work, gaining access to professional netball and community coaches was challenging. The questionnaire utilised was not subjected to reliability and validity testing whilst answers to Likert scale questions may have been influenced by central tendency bias, acquiescence bias or social desirability bias. We attempted to remove these forms of bias by ensuring completion of questionnaires online at players' and coaches’ convenience. Furthermore, collapsing Likert scales reduced the level of analytical details; however, as several comparative studies from other sports reported similar designs, this allowed ease of comparison between studies. Moreover, several potential confounding variables such as age, coaching experience and injury history may have influenced respondents’ perceptions.

## Conclusions

This study highlights limited knowledge of female athletes’ increased susceptibility to ACL injuries with a lack of communication and education of ACL injury prevention programs between sporting associations, coaches, and players. The results suggest a greater motivation among netball players and coaches to implement an ACL injury prevention program in netball. For those involved in ACL injury prevention programs, it is essential to understand how players and coaches perceive these programs to improve adherence and implementation of such programs. 

## Appendices

Appendix A

Links for Survey

ACL Injury Prevention Program in Welsh Netball: Knowledge, Implementation & Barriers amongst Coaches https://cardiffmet.eu.qualtrics.com/jfe/form/SV_cu6TyZVGzHWWnCRACL Injury Prevention Program in Welsh Netball: Knowledge, Implementation & Barriers amongst Players https://cardiffmet.eu.qualtrics.com/jfe/form/SV_b8BKCa9eHpAKBgh

Appendix B

**Table 6:** Stakeholders responsible for delivering ACL injury prevention programs ACL: Anterior cruciate ligament

	Players	Head Coach	Fitness Coach	Physiotherapist	Club Doctor	Sports Director
Players	1	2	3	22	0	0
Coaches	4	10	6	5	2	2

**Table 7:** Respondent’s perceptions of ACL injury susceptibility and injury seriousness ACL: Anterior cruciate ligament

Female athletes are at increased risk for sustaining ACL injury than male athletes			
	Agree	Neither Agree/Disagree	Disagree
Players	17	7	4
Coaches	15	12	2
Netball players are at a high risk of ACL injury			
	Agree	Neither Agree/Disagree	Disagree
Players	26	2	0
Coaches	28	1	0
ACL injuries can shorten a Netball player’s career			
	Agree	Neither Agree/Disagree	Disagree
Players	24	4	0
Coaches	27	1	1
ACL injuries can cause physical problems later in life			
	Agree	Neither Agree/Disagree	Disagree
Players	24	4	0
Coaches	26	3	0
ACL injuries have a negative impact on Netball player’s quality of life			
	Agree	Neither Agree/Disagree	Disagree
Players	24	3	1
Coaches	24	3	2
Participating with an ACL injury can have a negative impact on team performance			
	Agree	Neither Agree/Disagree	Disagree
Players	18	7	3
Coaches	21	6	2

**Table 8:** Players and coaches’ perceptions of ACL injury prevention programs ACL: Anterior cruciate ligament

It is possible to prevent some ACL injuries in netball			
	Agree	Neither Agree/Disagree	Disagree
Players	25	2	1
Coaches	22	7	0
Exercises scientifically proven to prevent ACL injuries should be performed by netball players			
	Agree	Neither Agree/Disagree	Disagree
Players	28	0	0
Coaches	29	0	0
Exercises scientifically proven to prevent ACL injuries should be incorporated into clubs’ training guidelines			
	Agree	Neither Agree/Disagree	Disagree
Players	28	0	0
Coaches	28	1	0
Exercises to prevent ACL injuries should be varied and progressed over time			
	Agree	Neither Agree/Disagree	Disagree
Players	26	0	2
Coaches	23	6	0
Exercises involving balance training can prevent ACL injuries			
	Agree	Neither Agree/Disagree	Disagree
Players	24	4	0
Coaches	25	3	1
Exercises involving controlled jumping and landing can prevent ACL injuries			
	Agree	Neither Agree/Disagree	Disagree
Players	26	2	0
Coaches	28	0	1
Exercises involving eccentric muscle strengthening can prevent ACL injuries			
	Agree	Neither Agree/Disagree	Disagree
Players	24	4	0
Coaches	19	10	0
Exercises involving cutting manoeuvres can prevent ACL injuries			
	Agree	Neither Agree/Disagree	Disagree
Players	23	3	2
Coaches	19	8	2
A warm-up jog/run can prevent ACL injuries			
	Agree	Neither Agree/Disagree	Disagree
Players	15	13	0
Coaches	14	8	7
A cool-down jog/run can prevent ACL injuries			
	Agree	Neither Agree/Disagree	Disagree
Players	13	13	2
Coaches	13	9	7

**Table 9:** Players' and coaches' experience with ACL injury prevention programs ACL: Anterior cruciate ligament; IPP: injury prevention program

Netball club has promoted awareness about risk of ACL injuries amongst Netball players, either in meetings, emails, or by other means			
	Agree	Neither Agree/Disagree	Disagree
Players	8	4	16
Coaches	8	9	12
Netball club has advocated use of an ACL IPP for my team, or directed me to resources to find information about such programs			
	Agree	Neither Agree/Disagree	Disagree
Players	8	4	16
Coaches	6	9	14
Netball coach/club representative has demonstrated how to perform an ACL IPP to me or my team			
	Agree	Neither Agree/Disagree	Disagree
Players	6	3	19
Coaches	4	7	13

**Table 10:** Implementation of ACL injury prevention programs ACL: Anterior cruciate ligament; IPP: injury prevention program

I would consider using IPP if it could be used in place of warm up			
	Agree	Neither Agree/Disagree	Disagree
Players	21	2	3
Coaches	15	3	5
The reason I do not use ACL IPP with my team is because I do not know the exercises			
	Agree	Neither Agree/Disagree	Disagree
Players	18	3	5
Coaches	19	3	1
I would consider using an ACL IPP if I could access information about the exercises			
	Agree	Neither Agree/Disagree	Disagree
Players	25	0	1
Coaches	22	1	0
I would consider using ACL IPP if somebody demonstrated the exercises properly			
	Agree	Neither Agree/Disagree	Disagree
Players	26	0	0
Coaches	22	1	0
